# Deceptive Body Movements Reverse Spatial Cueing in Soccer

**DOI:** 10.1371/journal.pone.0104290

**Published:** 2014-08-06

**Authors:** Michael J. Wright, Robin C. Jackson

**Affiliations:** 1 Centre for Cognition and Neuroimaging, Department of Psychology, Brunel University London, Uxbridge, United Kingdom; 2 Centre for Sports Medicine and Human Performance, Department of Sport and Exercise Science, Brunel University London, Uxbridge, United Kingdom; VU University Amsterdam, Netherlands

## Abstract

The purpose of the experiments was to analyse the spatial cueing effects of the movements of soccer players executing normal and deceptive (step-over) turns with the ball. Stimuli comprised normal resolution or point-light video clips of soccer players dribbling a football towards the observer then turning right or left with the ball. Clips were curtailed before or on the turn (−160, −80, 0 or +80 ms) to examine the time course of direction prediction and spatial cueing effects. Participants were divided into higher-skilled (HS) and lower-skilled (LS) groups according to soccer experience. In experiment 1, accuracy on full video clips was higher than on point-light but results followed the same overall pattern. Both HS and LS groups correctly identified direction on normal moves at all occlusion levels. For deceptive moves, LS participants were significantly worse than chance and HS participants were somewhat more accurate but nevertheless substantially impaired. In experiment 2, point-light clips were used to cue a lateral target. HS and LS groups showed faster reaction times to targets that were congruent with the direction of normal turns, and to targets incongruent with the direction of deceptive turns. The reversed cueing by deceptive moves coincided with earlier kinematic events than cueing by normal moves. It is concluded that the body kinematics of soccer players generate spatial cueing effects when viewed from an opponent's perspective. This could create a reaction time advantage when anticipating the direction of a normal move. A deceptive move is designed to turn this cueing advantage into a disadvantage. Acting on the basis of advance information, the presence of deceptive moves primes responses in the wrong direction, which may be only partly mitigated by delaying a response until veridical cues emerge.

## Introduction

A soccer player making a run with the ball will often execute a deceptive move known as a *step-over* in order to evade a defender. In recent decades, a number of international soccer players have become renowned for their skill in performing these moves, and awareness and appreciation of the step-over and its variants has increased amongst players, coaches and fans. This paper will examine how such deceptive moves produce their effects, and why even experienced players may be fooled by them.

Soccer is an example of an open-skill sport, that is, one where the environment and therefore the task at hand is constantly changing because of the actions of other players. Soccer players will benefit from being able to correctly predict the actions of their opponents because anticipation increases the time available for events such as intercepting a pass, blocking a shot at goal, or making a tackle. The importance, in interceptive sports, of body kinematics as a cue to future action, has been established in studies using point-light displays [1], [2]. Related studies have isolated which specific kinematic cues are predictive of the outcome of an action in sport, for example, the direction of a tennis serve [3], [4]. Furthermore, experiments within a range of interceptive sports, using video clips curtailed prior to critical events, consistently show better detection by experts of predictive information [2], [5], [6].

Competitive players also need to develop strategies to prevent their own actions from being anticipated. These strategies are broadly categorised as disguise or deception. Disguise strategies reduce the detectability of critical cues. For example, bowlers in cricket may minimize variations in different types of delivery such as topspinners and backspinners by keeping the movement pattern constant, except for the wrist action [7]. In the auditory modality, grunting by tennis players may be more than a vocal expression of effort, it may be an effective strategy in masking the sound of the racket-on-ball impact and thus degrading timing information [8].

Deception strategies introduce false or misleading cues that, if effective, result in the observer making an incorrect response [9]. Deceptive moves have been studied in rugby football [9]–[11], soccer [12]–[15] and tennis [16]. In these studies it was found that higher-skilled (HS) players are more accurate than lower-skilled (LS) players in predicting the outcome of deceptive moves, but that performance was nevertheless reduced by deception both for HS and LS players. This superior ability to anticipate both normal and deceptive action has been shown to depend on motor as well as sensory experience in the relevant sporting domain [17], [18].

When a visual cue directs attention to a visual location this has profound effects on the processing of information both at cued and uncued locations. The spatial cueing paradigm, whereby a visual cue stimulus can validly or invalidly indicate the location of a subsequent target, has given rise to many hundreds of experimental studies (see [19] for a review). A general finding is that reaction times (RTs) are faster for valid than for invalid cueing, so that the difference in RT provides an effective measure of spatial attention [20].

In a sport-related application of the spatial cueing paradigm [21], researchers studied directional judgements among novice basketball players. These experiments were based on the “head fake” in which a player attempts to deceive by gazing in one direction while passing or shooting the ball in another direction. Using stationary images of a basketball player about to pass the ball, the experiments demonstrated a spatial cueing effect of a player's head orientation, which produced a slowing of reaction times when incongruent with arm and body posture.

The use of stationary cues in spatial attention studies [14], [19] allows location and timing to be strictly controlled. On the other hand, Kibele [22] has proposed a cueing explanation for fast motor reactions in sports that is based on non-conscious analysis of perceived motion sequences of opponents. Could something as fluid and complex as a footballer's body movements act as an automatic spatial cue to the direction of play? A simpler case of cueing from biological motion is already known to occur: that is, the spatial cueing effect of point-light walker stimuli for left- or right- walking direction on detection of a brief visual target [23].

In the present study we investigate whether the kinematic body cues in typical one-on-one soccer play can produce a spatial cueing effect, and whether soccer expertise influences spatial cueing. The use of point-light stimuli allows the selective analysis of the effects of dynamical information, and we have tested the idea that the kinematics of a deceptive move reverses the spatial cueing effect.

## Materials and Methods

### Participants

Higher-skilled (HS) and lower-skilled (LS) male soccer players (age 18–33) were recruited by advertisement and personal contacts. HS players were defined as those currently belonging to a soccer club, training regularly, and competing in local or University leagues. LS participants comprised non-players or recreational players who did not train and did not play competitively. Demographic details and details of soccer experience were collected in a brief self-report questionnaire and are summarised in Table 1 for HS and LS participants; details of named clubs and leagues were checked online. Two different sets of participants undertook the two experiments.

**Table 1:** Soccer experience of the participant groups.

	Experiment 1		Experiment 2	
	Higher-skilled	Lower-skilled	Higher-skilled	Lower-skilled
	Mean (*SD*)	Mean (*SD*)	Mean (*SD*)	Mean (*SD*)
Age (years)	20.9 (2.9)	22.6 (4.6)	23.0 (3.7)	22.5 (3.3)
Years playing	13.1 (3.9)	2.8 (4.1)	12.2 (6.1)	6.4 (6.2)
Hours training/week	4.9 (2.8)	0.1 (0.45)	5.1 (3.6)	0.0 (0.0)

	*N*	*N*	*N*	*N*
Amateur league (FA charter)	15	0	7	0
British Universities & Colleges Sport league	10	0	10	0
Other local league	2	0	2	0
Not competing in a league	0	20	0	19
Total *N*	27	20	19	19

### Ethical Statement

The research was approved by the Psychology Research Ethics Committee of Brunel University before initiating the study. It followed British Psychological Society guidelines for research with human participants, in accordance with the Declaration of Helsinki. Written informed consent was obtained from all participants prior to taking part, and they were informed of the right to withdraw from the study.

### Stimuli

Video clips (720×576 pixels, 24 bit colour) were made of three right-footed junior international level soccer players dribbling the ball towards a video camera (Panasonic NV GS400 recording at 25 frames/second) placed at a distance of 11.5 meters from the start of the player's run, in an indoor sports hall. The actors ran directly towards the camera, then on passing a floor marker, moved obliquely to the left or right as they would in evading a defending player's interception. The actors also performed a step-over in 50% of runs immediately prior to a direction change. The colour video was edited frame by frame to produce sparse binary (black/white) point-light representations consisting of 18 small disc markers on principal body joints and extremities (forehead, chin, L shoulder, R shoulder, L elbow, R. elbow, L wrist, R wrist, L pelvis, R pelvis, L knee, R knee, L ankle, R ankle, L toe, R toe, L heel, R heel). The ball was represented by a white disc matched to the silhouette of the ball in each frame, thus increasing in size as the player approached the camera. There was no representation of surface texture, depth, orientation or colour, either that of the player or that of the background in the point-light video. Occluded dots were masked. It is significant for the interpretation of results that the point-light player's head was represented by only two dots, one on the centre forehead and one on the chin. A sequence of stopped-motion frames from the point-light video of a step-over move is shown in Figure S1.

To generate the different levels of temporal occlusion, 6 normal and 6 deceptive source videos (two normal and two deceptive from each player) were cut off at various time points relative to the passing of the floor marker yielding 48 clips. Four occlusion levels were used relative to the final frame before the foot either made contact with (non-deceptive condition) or passed in front of (deceptive condition) the ball (−160, −80, 0 and +80 ms). In experiment 1, clips were presented in a pseudo-random order using Windows Media Player (Microsoft Inc.), with a 4 s blank interstimulus interval for responses. In experiment 2, stimuli were presented in random order and corresponding keyboard responses were recorded using e-prime2 (Psychology Software Tools, Inc.). They were viewed on a 43 cm computer screen at 120 cm viewing distance, and the players subtended approximately 7 deg vertically on closest approach. The point-light video was used in experiments 1 and 2, and the colour video was used for comparative purposes in experiment 1.

### Design

The first experiment followed the design that is conventional for studying the effects of temporal occlusion on the ability to predict the outcome of an opponent's moves in a sport-related anticipation task. The purpose of this experiment was to compare the effects of low-resolution kinematic displays versus full video when HS and LS participants predict the direction of normal and deceptive soccer moves. The full video and point-light stimuli were presented in blocks, with order counterbalanced across participants; occlusion and deception variables were randomised within blocks, and the dependent measure was accuracy in predicting the final direction of the ball. The second experiment utilised the point-light video clips in the role of a spatial prime, and measured the reaction time (RT) to subsequent targets presented congruent or incongruent with the future trajectory of the ball. The purpose of this experiment was to establish whether body kinematic stimuli produce spatial cueing effects, whether deceptive primes produce reversed spatial cueing, and whether there are expertise differences in the normal and reversed cueing effects.

### Procedures

Participants for experiment 1 were shown example clips of late-occluded video slides representing normal video with and without step-over, and point-light video with and without step-over, each with a brief identification from the experimenter. They were then instructed as follows: “Your task is to predict the direction that the player will take the ball, and you should respond as accurately as possible. To make the task more challenging, most of the clips are cut off before the ball changes direction. Please respond after every video clip, choosing the more likely alternative if you are unsure. If you think he took the ball to your right, say “right”. If you think he took the ball to your left, say “left”.”

In experiment 2, the aim was to assess the effect of the video clips on a subsequent spatial target in the absence of any explicit interpretation or decision made on the video itself. Participants were shown example clips of the point-light player and the target stimuli. They were told that the soccer clips included both normal and deceptive (step-over) moves, but was not informative about the side of the target. For the experiment itself, on-screen instructions asked them to watch the video clips but respond only to the target. They were asked to press the “9” key with their right hand if the target was on the right and the “1 key with their left hand if the target was on the left. They were asked to respond as quickly and as accurately as possible.

## Results

### Experiment 1

Prediction of ball direction in normal and deceptive soccer moves using full video and point-light displays.

**Figure 1:**
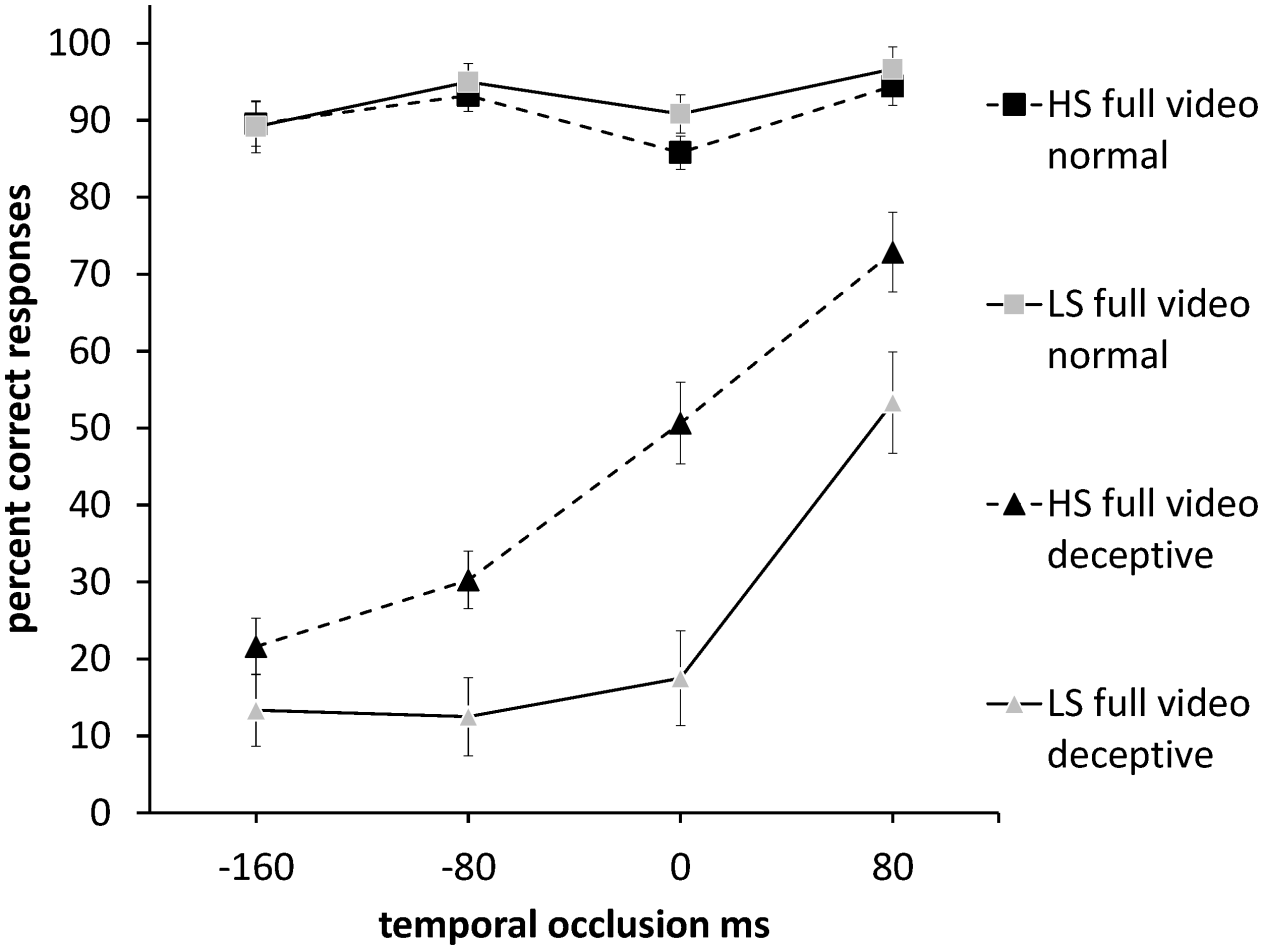
Accuracy in predicting the direction of the ball in normal and deceptive full colour soccer video clips in HS and LS players. Chance accuracy is 50%. The x-axis shows the temporal cut-off of the soccer video. Squares represent normal moves and triangles represent deceptive moves. Black symbols indicate HS players, and grey symbols LS players. Error bars are ± 1 SEM.

**Figure 2:**
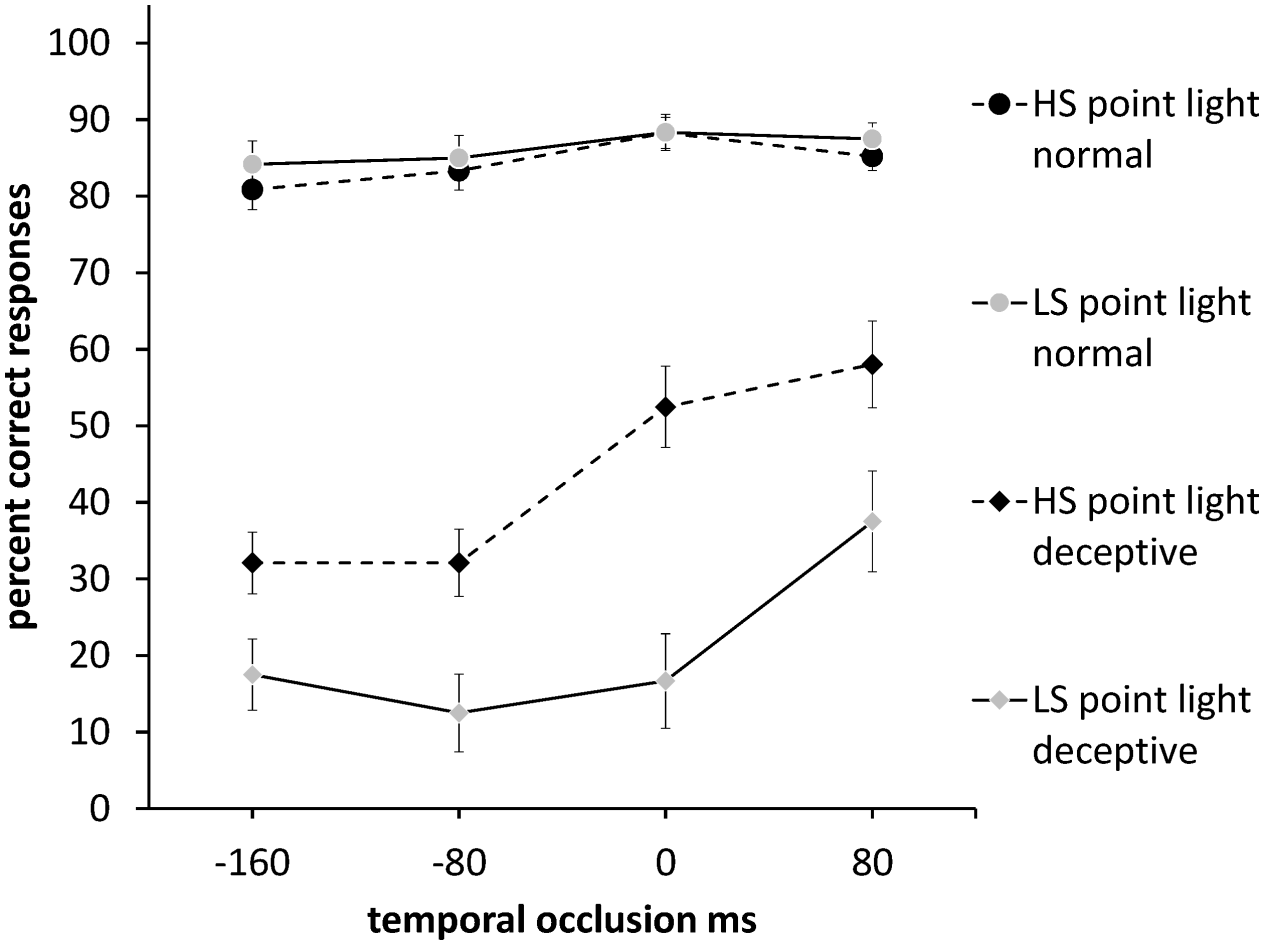
Accuracy in predicting the direction of the ball in normal and deceptive point-light soccer video clips in HS and LS players. Chance accuracy is 50%. The x-axis shows the temporal cut-off of the soccer video. Squares represent normal moves and triangles deceptive moves. Black symbols indicate HS players, and grey symbols LS players. Error bars are ± 1 SEM.

Twenty-seven higher-skilled (HS: *M* = 20.9, *SD* = 2.9 years of age) and 20 lower-skilled (LS: *M* = 22.6, *SD* = 4.5 years of age) soccer players viewed 48×2 s full video and 48×2 s point-light video clips and indicated the direction required for interception of a move (observer's left or right, forced choice). The dependent measure was percentage accuracy in judgment of direction. Expertise (HS, LS) was a between-participant variable, and stimulus format (full colour video, point-light), deception (normal, deceptive) and occlusion (−160 ms, −80 ms, 0 ms, +80 ms) were within-participant variables. Mauchly's test revealed a significant departure from sphericity for Occlusion (*W* = .63, *p*<.005) and Occlusion×Deception (*W* = .60, *p*<.0005), so a Greenhouse-Geisser correction was employed for these effects. Other data requirements for ANOVA were met. Partial eta squared (η^2^_p_) was taken as the measure of effect size appropriate for a mixed ANOVA [24]. Figures 1 and 2 summarise expertise effects, and the effects of temporal occlusion, video type, and deception. The corresponding statistical results from ANOVA are presented in Table 2.

**Table 2:** ANOVA results for experiment 1.

factor	*F*	*df*	*p*	η^2^_p_
*Expertise*	17.2	1,45	<.0005	.28
*Video type*	8.85	1,45	<.005	.16
*Deception*	506.1	1,45	<.0001	.92
*Occlusion*	42.5	2.3,102.6	<.0005	.49
*Expertise×Deception*	22.1	1,45	<.0005	.33
*Video type×Occlusion*	8.33	3,135	<.0005	.16
*Deception×Occlusion*	30.1	2.3,101.2	<.0005	.40
*Expertise×Deception×Occlusion*	3.66	2.3,101.2	<.05	.08
*Video type×Deception×Occlusion*	4.22	3,135	<.01	.09
*All other interactions*			n.s.	

Over all conditions, HS participants (*M* = 65.7%, *SD* = 8.82%) were more accurate than LS (*M* = 56.1%, *SD* = 6.2%). Differences were greater on deceptive moves (HS: *M* = 43.8%, *SD* = 15.5%; LS: *M* = 22.6%, *SD* = 13.8%) than on normal moves (HS: *M* = 87.6%, SD = 6.8%; LS: *M* = 89.6%, *SD* = 6.0%). Other significant effects of expertise such as a three-way interaction with occlusion and deception suggest that there are differences in the way that HS and LS participants processed cues. Significant within-group effects included a main effect of video type, with higher overall accuracy on full video (*M* = 62.9%, *SD* = 8.8%) than point-light (*M* = 58.8%, *SD* = 11.5%) stimuli. The largest effect size (η^2^_p_ = .92) was found for the overall difference in accuracy between normal (*M* = 88.6%, SD = 6.5%) and deceptive (*M* = 32.2%, *SD* = 18.1%) moves. The variations in the shapes of the temporal occlusion curves in Figures 1 and 2 reflect significant interactions of temporal occlusion with video type and deception (Table 2) which would thus be consistent with variations in the pick-up of cues over time for the different video types and move types.

Planned comparisons were used to analyse the performance of HS and LS players on deceptive and normal moves, relative to a chance level of performance. As Table 3 shows, for normal moves, accuracy was better than chance at all occlusion levels for both higher-skilled and lower-skilled groups in both full video and point-light formats (one-sample t-tests). However, for deceptive moves, in both display conditions performance was below chance for early occlusion levels (LS: −160, −80, 0; HS: −160, −80 ms) and not significantly different from chance at late occlusion levels (LS: 80; HS: 0 ms). Performance at 80 ms for the higher-skilled group was also at chance in the point-light condition and was significantly above chance in the full video condition. Thus, the deceptive move successfully biased anticipation of ball direction in the wrong direction for both higher-skilled and lower-skilled groups, but higher-skilled players were able to show compensation particularly on late occluded clips (Figures 1, 2, Table 3).

**Table 3:** Accuracy of direction prediction compared with chance performance: entries in bold represent performance significantly below chance.

	lower-skilled			higher-skilled		
Full video	*t*	*df*	*p*	*t*	*df*	*p*
Normal −160 ms	10.1	19	<.0005	15.6	26	<.0005
Normal −80 ms	21.1	19	<.0005	19.4	26	<.0005
Normal 0 ms	15.9	19	<.0005	16.9	26	<.0005
Normal +80 ms	30.5	19	<.0005	14.4	26	<.0005
Deceptive −160 ms	**−8.5**	**19**	**<.005**	**−7.8**	**26**	**<.005**
Deceptive −80 ms	**−9.4**	**19**	**<.0005**	**−5.0**	**26**	**<.005**
Deceptive 0 ms	**−6.1**	**19**	**<.005**	.11	26	n.s.
Deceptive +80 ms	.56	19	n.s.	4.4	26	<.005

Point-light	*t*	*df*	*p*	*t*	*df*	*p*
Normal −160 ms	10.3	19	<.0005	12.5	26	<.0005
Normal −80 ms	13.1	19	<.0005	12.5	26	<.0005
Normal 0 ms	18.0	19	<.0005	17.8	26	<.0005
Normal +80 ms	22.6	19	<.0005	17.1	26	<.0005
Deceptive −160 ms	**−7.1**	**19**	**<.005**	**−4.4**	**26**	**<.005**
Deceptive −80 ms	**−11.8**	**19**	**<.0005**	**−3.4**	**26**	**<.05**
Deceptive 0 ms	**−6.5**	**19**	**<.005**	.69	26	n.s.
Deceptive +80 ms	−2.5	19	n.s.	.20	26	n.s.

Significance levels are based on planned comparisons against chance with one-sample t-tests, two-tailed, Bonferroni corrected.

The results showed a similar overall pattern of results for full video and point-light stimuli, but with superior performance on full video consistent with the reduction of spatial resolution and pictorial depth cues in the point-light kinematic representation. The performance of HS was superior to LS participants on deceptive trials, this corresponding to the significant interaction between expertise and deception in ANOVA. The fact that deceptive moves gave significant effects in the opposite direction to the veridical ball direction suggests that the deceptive move may cause a reversal of a spatial attention-biasing effect. This is tested in the next experiment.

### Experiment 2

Spatial cueing by normal and deceptive soccer moves in higher-skilled and lower-skilled groups.

**Figure 3:**
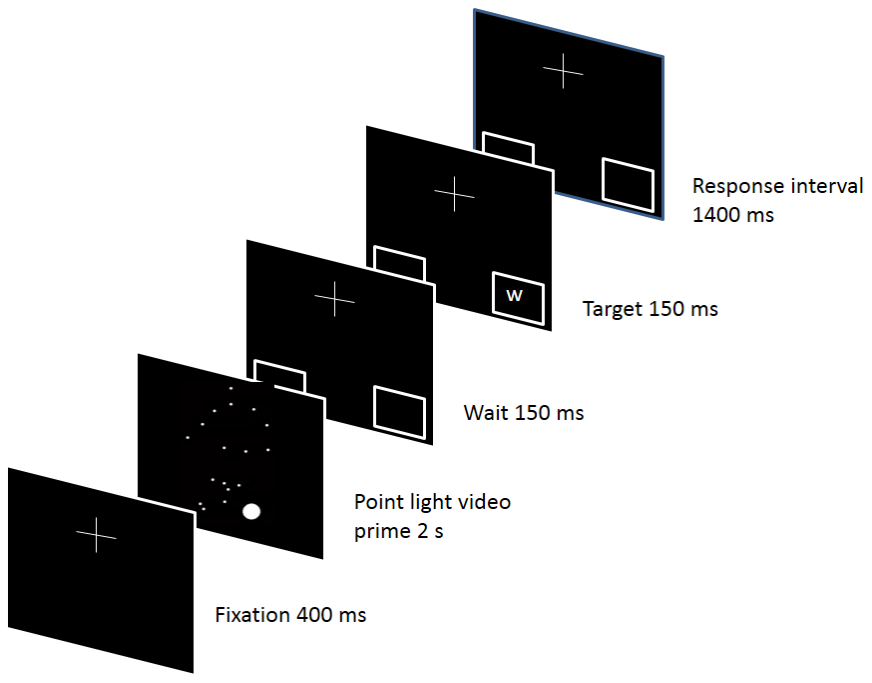
Stimulus sequence for a single trial of Experiment 2.

In the second experiment, there were 19 higher-skilled males (HS: *M* = 23.0, *SD* = 3.7 years of age) and 19 lower-skilled males (LS: *M* = 22.5, *SD* = 3.3 years of age). The criteria for allocating participants to groups were the same as in Experiment 1, but no participants undertook both experiments. The cueing stimuli were identical with the point-light test set in Experiment 1, and each stimulus condition was presented twice, making 96 randomised trials. The stimulus sequence for a single trial is shown in Figure 3. The target stimulus was a small (0.5 deg) letter placed within a square box. The target letter could appear either in the left hand or in the right hand box on each trial. The onset of the target letter was 150 ms after the end of the video clip, and the duration was 150 ms. Instructions to participants indicated that they should watch the footballer but respond only to the target. The response required was a corresponding left-handed (key = 1) or right-handed (key = 9) keypress. Following the keypress there was a delay of 400 ms before the onset of the next video clip.

Mean errors (missed and incorrect responses to the target) ranged between 2.7%–3.7%, and were omitted from the analysis. All RT distributions departed significantly from normality (Kolmolgorov – Smirnov test, all *p*<.05). However, since the criterion for a cueing effect is a difference in RT between congruent and incongruent trials, the mean difference (RT incongruent – RT congruent) was computed for each within-participant variable (two levels of deception and four levels of occlusion). These cueing RT differences were consistent with a normal distribution for all eight variables (Kolmolgorov – Smirnov test, all n.s.) and Mauchly's test indicated no significant departures from sphericity. ANOVA was therefore conducted with the cueing RT differences as dependent variable, with all data requirements for ANOVA being met. The within subjects factors were deception (deceptive, normal) and occlusion (−160, −80, 0 and +80 ms temporal occlusion). The between-participant variable was expertise (lower-skilled, higher-skilled).

**Figure 4:**
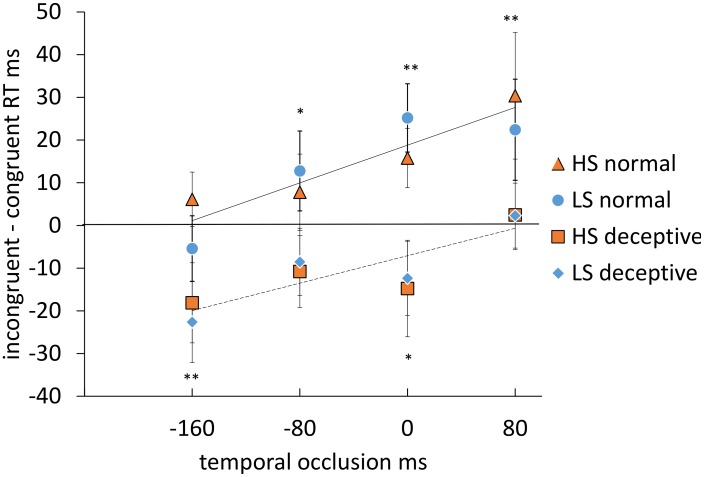
Cueing RT (incongruent minus congruent RT) is shown as a function of temporal occlusion for two types of cueing stimulus (normal and deceptive football moves) and two groups of participants (HS and LS). Regression lines were fitted to mean data by a least squares method (normal, solid line and deceptive, dashed line). One-sample t-tests show, for normal and deceptive moves, at which occlusion levels the mean cueing RT (across all participants) is significantly different from zero at *p*<.05 *, *p*<.01 **. Error bars are ± 1 SEM.

Normal soccer moves gave shorter reaction times (RTs) when congruent with the target, and deceptive soccer moves gave shorter RTs when incongruent with the target, that is the latter gave rise to a reversed cueing effect. The strongest effect observed on the incongruent – congruent RT difference was thus a significant main effect of deception, *F* (1, 36) = 19.22, *p*<.0005, η_p_^2^ = .35. As the graph in Figure 4 shows, cueing RT was generally positive for normal moves and negative for deceptive moves. This means that for normal moves, when the target was congruent with the direction of play, RTs were shorter. Conversely, for deceptive moves, RTs were shorter when the target was incongruent with the direction of play, but congruent with the direction of the feint or bluff move. There was also a significant main effect of occlusion, *F*(3, 108) = 6.03, *p*<.005, η_p_^2^ = .14. There were no significant differences due to expertise, and there were no other significant main effects or interactions. The direction of the occlusion effects was tested using polynomial contrasts, and these showed a linear relationship between cueing RT and occlusion level over the measured range, such that cueing RT becomes more positive with decreasing occlusion, *F*(1,36) = 16.72, *p*<.0005. η_p_^2^ = .32. Thus, for normal moves, increasing information from the video led to more normal cueing, that is, a bigger advantage for congruent moves. For deceptive moves, increasing information from the video led to a smaller disadvantage for congruent moves, that is a decreasing deceptive effect (Figure 4).

This implies that the critical cues that constitute the normal and deceptive moves had a different time-course, with the deceptive cues being earlier. Thus, rather than being identical up to the point the foot contacts or passes in front of the ball, the player's motion up to −160 ms is already generating a reverse cueing effect in the deceptive condition. Conversely, at this point there is no cueing effect for normal trials.

## Discussion

The findings show that amateur soccer players in University and local league competitions are better than inexperienced or recreational players in predicting the direction of play. However both higher-skilled and lower-skilled players were adversely affected by deceptive moves, with LS significantly choosing the wrong direction at all levels of temporal occlusion, and HS significantly choosing the wrong direction for early-occluded sequences, and performing at or above chance level for late-occluded sequences. These results are broadly in line with previous findings in rugby football [9], [10] and tennis [16]. The first experiment showed that the effects of deception and expertise were similar in full video and point-light versions of the stimuli, although there was evidence of greater pickup of task-relevant cues from the full video stimuli. The expertise effects in Experiment 1 were not due to the verbal response mode because equivalent effects were found with manual response for full video [14] and point light [15] stimuli. The primary reason for conducting the first experiment was to establish expertise differences in an independent sample from the same recruitment pool and over the same range of temporal occlusion as the main (second) experiment and to establish the adequacy of the point-light stimuli for demonstrating effects of deceptive moves in comparison with full video stimuli.

Caution must be exercised in extrapolating from these results to actual soccer play, because of concerns with ecological validity [25]. The angular size of the footballer stimuli on the computer screen was such that all the action was comfortably contained within the video frame, and was considerably smaller than would occur in a close interception on the pitch. The video stimuli were substantially impoverished compared with immersive 3D live action on the soccer field, and the element of sensory-motor interaction was absent. It is likely that higher-skilled players would be able to benefit from experience of these richer dynamic and contextual cues [26]. However the strengths of the reductive approach are that it allows isolation and analysis of specific cues, in particular, kinematic cues, and that it adds to a growing literature on biological stimuli as spatial cues.

An important development in understanding spatial attention is the evidence that a range of socially relevant stimuli can function as spatial cues. Both people within a scene and eyes within a face attract a strong fixation preference [27]–[29]. When we observe another person whose eyes are fixated on an object, our attention is directed towards that object. Direction of gaze acts as a spatial cue both in tightly-controlled laboratory experiments and in naturalistic scenarios [30], [31]. Social attention cues other than gaze have been less thoroughly studied but eye direction, head direction and hand gestures are all important components in directing social attention [32]. Gaze cues can also misdirect attention and this is one means whereby magicians create their tricks and illusions [33]. Although the walking direction of a point-light walker towards the left or right can produce a spatial cueing effect in the direction of motion [23], in the present experiment, cueing to a lateral target occurred when the net direction of locomotion of the point-light footballer was towards the observer. Kinematic cues can signal a future change of direction, and the step-over may resemble a mirror-image of the honest kinematic cues that are predictive of the direction of the turn. Thus in the present study, misdirection of spatial attention is proposed as the mechanism whereby deceptive soccer moves produce their effect.

Results indicated that the effects of normal and deceptive movements can be produced with purely kinematic, low-resolution, point-light information. Because the head was represented by only two vertical dots, these effects do not rely on direction of gaze. Furthermore, the cueing experiment indicated that in normal moves, spatial attention for targets in the future direction of ball play was cued by the player's body kinematics, whereas for deceptive moves, spatial attention was cued for targets on the opposite side to the future direction of ball play. It was shown that the time course of the reversed spatial priming in deceptive moves was earlier than the normal spatial priming in normal moves. This is consistent with the time-course and primary direction of the deceptive (step-over) and honest (outer foot pushing ball) movements.

Figure 4 showed that there was also a sustained cueing deficit for deceptive moves compared with normal moves across the whole −160 to +80 ms range. The dynamics of the deceptive move have a reversed spatial cueing effect like that of a normal move but in the wrong direction. The presence of deceptive moves thus opposes the overall attention cueing advantage that occurs with normal moves.

The present results exemplify the function of body kinematics as a cue for directing social attention; a function that is pre-eminent in dual interactions. One may suppose that the cueing of spatial attention by observed body kinematics is likely to be an evolutionary adaptation present in other mobile, visually proficient species. Cueing of spatial attention by body kinematics would help predators to anticipate the movements of prey, and equally it is advantageous for prey species to provide incongruent kinematic cues to evade predators. A good example of this is when wild mice execute sudden 90 degree turns [34]. Indeed spatial cueing by body kinematics may play a role in a wide range of within-species and inter-species behaviours that are reciprocal and time-critical.

In soccer, an advantage of acquiring deceptive skills such as the step-over is suggested by the current results. Not only can such moves frequently reverse the predictive decisions of opponents (Experiment 1), they can do so for HS as well as LS observers. On normal moves, HS and LS observers performed with similar levels of accuracy. On deceptive moves both showed high error rates relative to normal moves, but the HS observers were more accurate than LS, particularly on late-occluded stimuli. However, the spatial cueing effects on RT that occur in the absence of explicit identification of direction of play did not show any significant difference between HS and LS players. The different pattern of expertise effects in the two experiments could be explained by task differences, because the two tasks engage different attentional processes.

Evidently the spatial cueing effects of normal and deceptive movements in Experiment 2 are automatic, like those produced by arrow cues [19] gaze cues [31] or other biological motion cues [23] since they occur when the cue is not predictive of the target, and where instructions are given not to process the cue. The cueing effect on normal moves had the effect of speeding reaction times when the direction of the turn was congruent with the side of the target, and cue strength increased as more of the action was revealed with decreasing temporal occlusion. On deceptive moves, a reverse cueing effect occurred which had the effect of slowing reaction times to stimuli in the direction of the turn, relative to the incorrect direction. The reverse cueing effect decreased as more of the action was revealed with decreasing temporal occlusion, implying that the kinematics of the earlier parts of the deceptive move were more effective.

Thus correct prediction of the direction of a deceptive move requires a decision counter to an automatic adverse attention-biasing effect, and HS players achieved this more frequently than LS players in Experiment 1. This may require the move to be explicitly recognised as deceptive. In support of this view, HS players were found to be better than LS players at differentiating between normal and deceptive moves [15]. Secondly, it was found that the processing of deceptive moves recruited brain areas that are associated with error correction and inhibitory control, of which an example would be inhibiting an incorrect response tendency to a deceptive move [14]. Thirdly it would follow from this that for direction judgments in the presence of deception, accuracy should be increased by delaying a response, and that HS players should thus paradoxically have slower reaction times than LS, as has been found in the case of rugby sidesteps [10]. To the extent that HS players are able to correct, on some trials, an automatic response tendency based on spatial cueing by body kinematics, this could help account for the superior accuracy of HS players over LS players in direction prediction for deceptive moves.

## Conclusions

Higher-skilled and lower-skilled soccer players were similar in accuracy of predicting the direction of play in normal and point-light soccer video clips, but both were inaccurate in direction prediction of deceptive moves. In relative terms, higher-skilled players were more accurate than lower-skilled players whose significantly below-chance accuracy, except at the latest occlusion level, implies a profound susceptibility to deception.

Occluded point-light video clips were shown to produce spatial cueing effects, thus body kinematics can direct attention in a predictive fashion. Reaction time to a small lateral letter target was faster when the side of the target corresponded with the occluded final direction of the soccer move, but only when it was a normal move. When it was a deceptive move, reaction time was faster when the target was incongruent with the (occluded) final direction of the move. Deceptive soccer moves produce reversed spatial cueing. The time course of normal and reverse cueing effects was consistent with the kinematics of the step-over (deceptive) and normal (honest) movements.

## Supporting Information

Figure S1 **Kinematics of a deceptive soccer move sampled at 80 ms intervals (left to right, top to bottom).** The step-over begins in the top row. The player's right foot passes in front of the ball between the second and third frames of the middle row (marked with an x) then touches the ground and takes the player's weight. The bottom row shows the subsequent push with the player's left leg, and the left foot finally makes contact with the ball in the final frame, sending the ball to the player's left.
(TIFF)

Data S1 **Individual participants' data and results for Experiment 1.**
(XLSX)

Data S2 **Individual participants' data and results for Experiment 2.**
(XLSX)
