# Supplementary figures and images for: Deceptive Body Movements Reverse Spatial Cueing in Soccer
Source: PLoS One. 2014 Aug 6;9(8):e104290. doi: 10.1371/journal.pone.0104290 (PMC4123942; doi:10.1371/journal.pone.0104290)

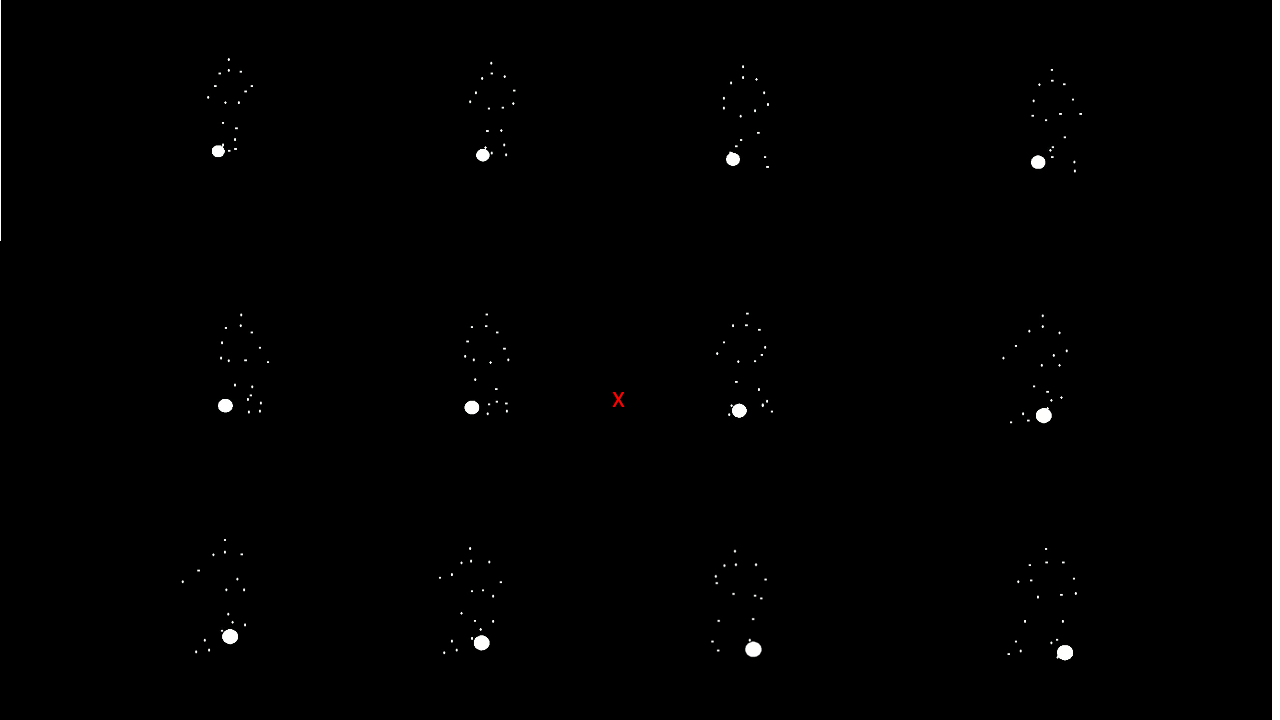

Supplement: Figure S1 — Kinematics of a deceptive soccer move sampled at 80 ms intervals (left to right, top to bottom). The step-over begins in the top row. The player's right foot passes in front of the ball between the second and third frames of the middle row (marked with an x) then touches the ground and takes the player's weight. The bottom row shows the subsequent push with the player's left leg, and the left foot finally makes contact with the ball in the final frame, sending the ball to the player's left. (TIFF) [file pone.0104290.s001.tiff]
